# Supplementary material for: Transient drug-tolerance and permanent drug-resistance rely on the trehalose-catalytic shift in Mycobacterium tuberculosis
Source: Nat Commun. 2019 Jul 2;10:2928. doi: 10.1038/s41467-019-10975-7 (PMC6606615; doi:10.1038/s41467-019-10975-7)
Supplement: Supplementary file 1 — Supplementary Information [file 41467_2019_10975_MOESM1_ESM.pdf]

Supplementary Information

Transient drug-tolerance and permanent drug-resistance rely on the trehalose-catalytic shift in *Mycobacterium tuberculosis*

Lee *et al*.

This document contains Supplementary Figures 1 – 12, Supplementary Figure Legends 1 – 12, and Supplementary Tables 1 – 2.

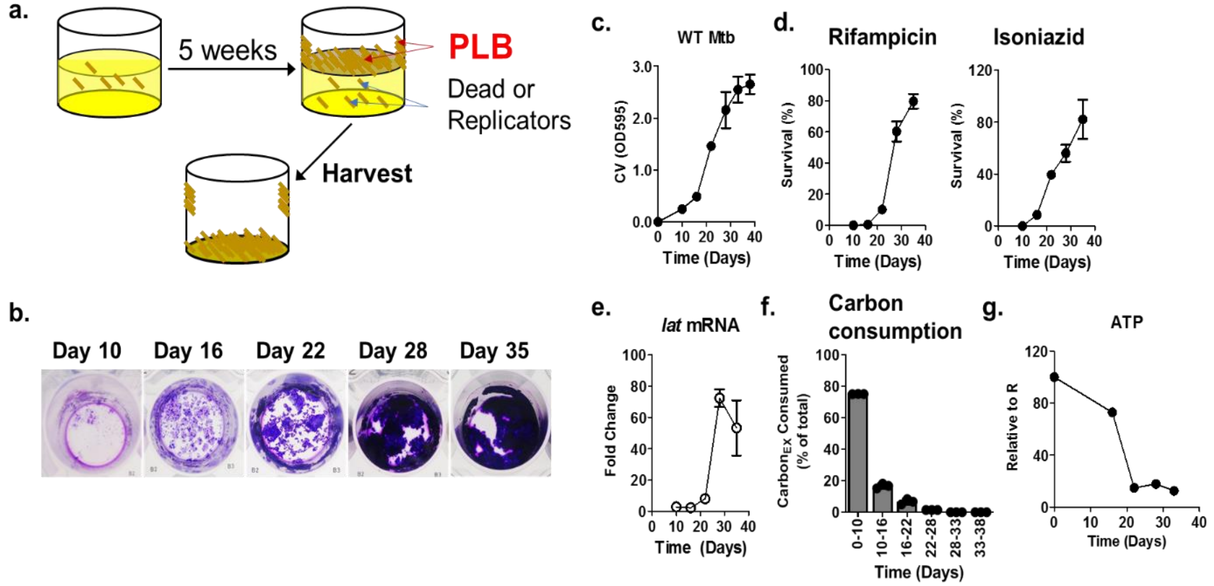

Supplementary Figure 1. Characterization of *in vitro* mycobacterial biofilm culture system.

a. Mycobacterial biofilms were cultured in 24-well microplates using Sauton nutrient-minimal media lacking detergent with limited gas exchange. Following a 5-week incubation, Mtb persister-like bacilli (PLB) formed at the wall-media border and air-media interface. PLB were selectively harvested using needle-syringes to remove both precipitated and floating bacilli. This is created by the authors. A time-course enrichment of the PLB in mycobacterial biofilm culture was monitored by **b**. crystal violet (CV) staining at days 10, 16, 22, 28, and 35, and **c.** measuring CV staining by OD_{595}. **d**. Levels of drug-tolerance against both rifampicin and isoniazid, **e**. mRNA expression of *lat*, an Mtb persister transcriptional biomarker, **f**. external carbon uptake and/or consumption using universally ^{13}C labeled [U-^{13}C] glycerol, and **g**. intrabacterial ATP levels were monitored as PLB grew and matured within the mycobacterial biofilm culture. All values are average of experimental triplicates ± s.e.m.

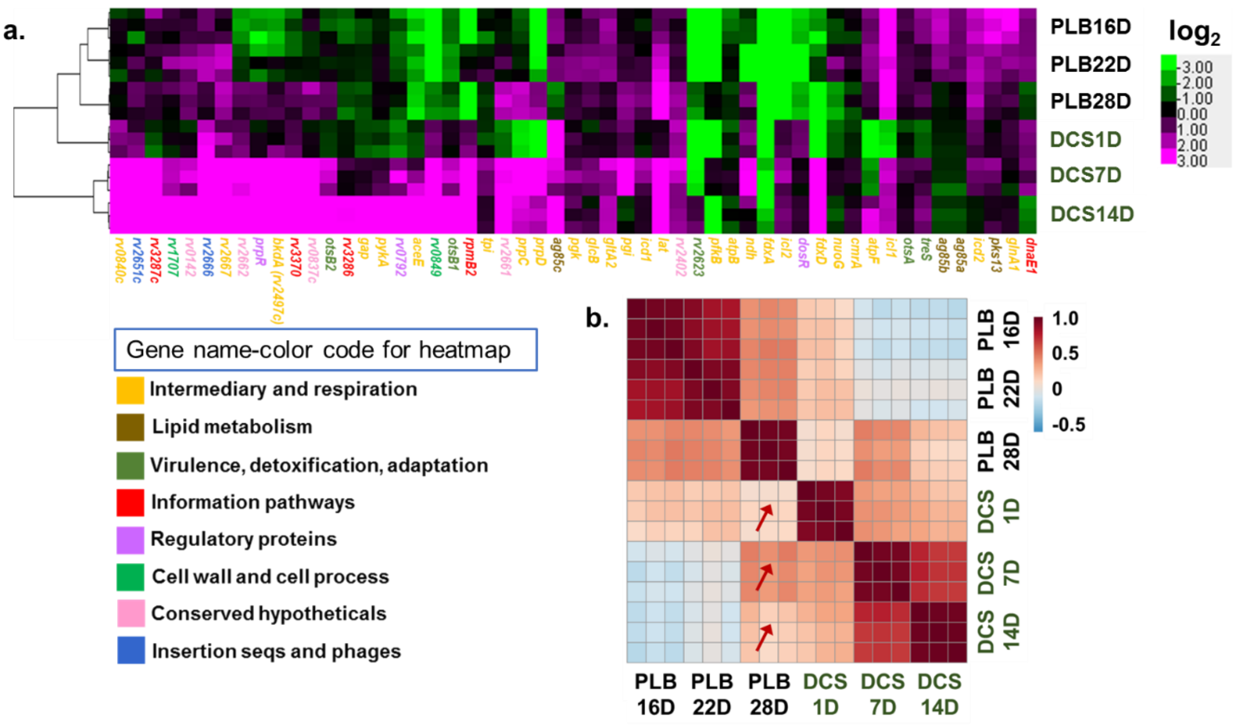

Supplementary Figure 2. Transcriptional correlation between PLB and Mtb persisters.

a. Hierarchical clustering of 54 genes (columns) in all conditions (rows), parsed using the image generation program treeview (http://jtreeview.sourceforge.net/). Data are depicted on a $\log_2$ scale relative to *sigA* transcripts. The conditions included: PLB harvested at days 16, 22, and 28 of biofilm culture (PLB16D, PLB22D, and PLB28D) and Mtb persisters harvested at days 1, 7 and 14 after treatment with 100 µg/mL d-cycloserine (DCS1D, DCS7D, and DCS14D). 54 genes were selected among those involved in intermediary/respiration, lipid metabolism, virulence/detoxification/adaptation, information pathways, regulatory proteins, cell wall/cell process/ conserved hypotheticals, and insertion seqs/phages.

b. The correlation heatmap displays the correlation coefficients (Spearman) among 54 genes that were selected. On the color-coded scale (right), red indicates a positive correlation and blue indicates a negative correlation. Red arrows indicate the positive correlation of transcriptional remodeling between PLB28D and DCS harvested at all time-points.

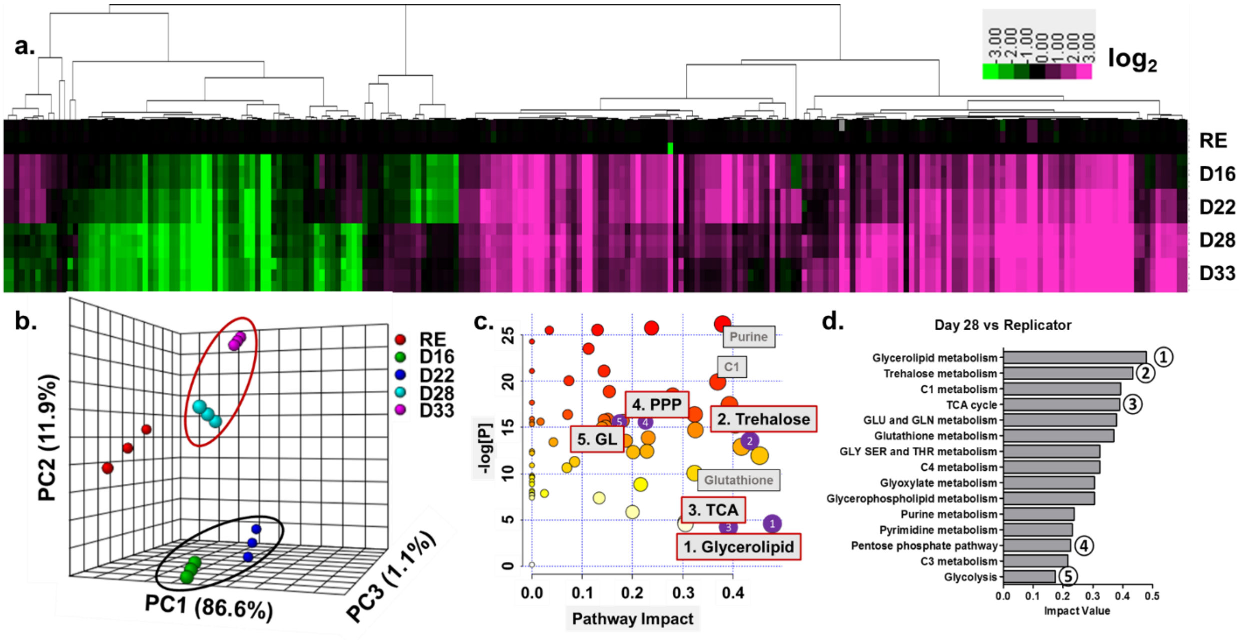

Supplementary Figure 3. Pathway mapping using the PLB metabolome in a time-dependent manner.

a. Clustered heatmap depicting levels of ~260 metabolites of PLB harvested at days 16, 22, 28, and 35 of the mycobacterial biofilm system and replicating state (RE). Rows depict harvesting time of replicators (RE) and PLB from day 16 (D16) to day 35 (D35). Columns indicate individual metabolites. Data were parsed using uncentered Pearson's correlation with centroid linkage clustering and rendered using the image generation program treeview. Data are depicted on a $\log_2$ scale relative to replicating state. Source data are provided as a Source Data file.

b. Principal component analysis (PCA) using PLB metabolome in a time-dependent manner. Three dimensional PCA score plots reveal separation in metabolite profiles induced by different stages of PLB formation. Black circle includes D16 and D22 PLB metabolome and red circle includes D28 and D35 PLB metabolome. Both are different from replicating state metabolome (RE).

c. Statistically over-represented pathways in PLB at day 28 (D28) relative to Mtb in a replicating state (RE) were screened by log[P] values (Y-axis) and pathway impact scores (X-axis) and **d**. pathway impact ranks of day 28 PLB were described. Trehalose metabolism and glycerolipid metabolism belong to top-ranked pathways and central carbon metabolism including glycolysis, the pentose phosphate pathway, and the TCA cycle belong to high-ranked pathways.

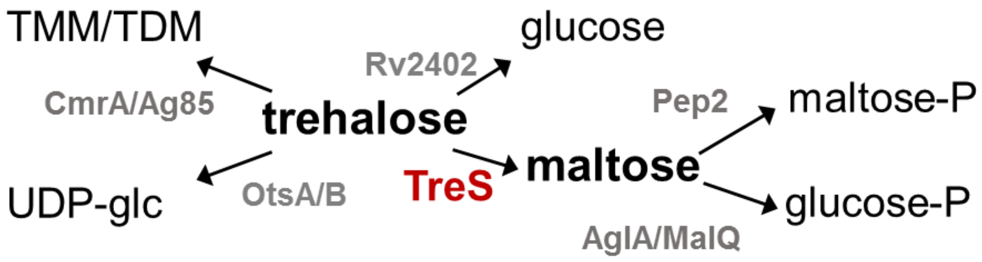

Supplementary Figure 4. Metabolites and genes involved in Mtb trehalose metabolism pathways.

Mtb has multiple trehalose metabolism pathways including OtsA/B, Rv2402, CmrA/Ag85s, and TreS. OtsA/B involves trehalose biosynthetic activity using uracil diphosphate-glucose (UDP-glc). CmrA and Ag85s involve the biosynthesis of TMM (trehalose monomycolate) and TDM (trehalose dimycolate), respectively. Rv2402 is annotated to be involved in trehalose dissociation to produce two molecules of glucose. TreS mediates the interconversion between trehalose and maltose. Pep2 encodes maltose kinase that phosphorylates maltose to produce maltose phosphate. Maltose also serves as a substrate for the biosynthesis of glucose-P by AglA and MalQ. Black font depicts metabolite names and gray font depicts catalytic enzyme names.

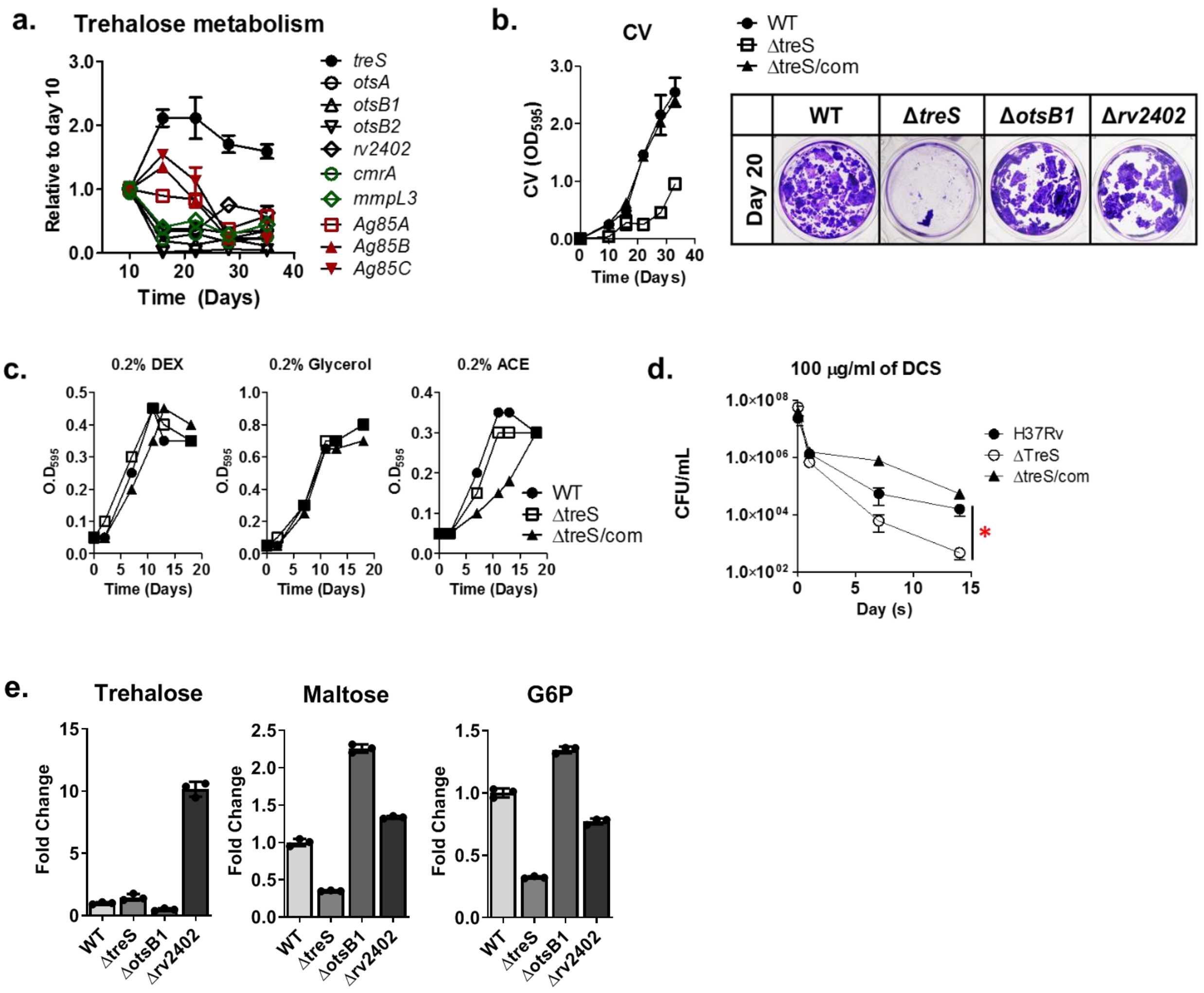
a.
Trehalose metabolism
Relative to day 10
Time (Days)
treS
otsA
otsB1
otsB2
rv2402
cmrA
mmpL3
Ag85A
Ag85B
Ag85C
b.
CV
CV (OD595)
Time (Days)
WT
ΔtreS
ΔtreS/com
WT
ΔtreS
ΔotsB1
Δrv2402
Day 20
c.
0.2% DEX
0.2% Glycerol
0.2% ACE
O.D595
Time (Days)
WT
ΔtreS
ΔtreS/com
d.
100 μg/ml of DCS
CFU/mL
Day (s)
H37Rv
ΔTreS
ΔtreS/com
*
e.
Trehalose
Maltose
G6P
Fold Change
WT
ΔtreS
ΔotsB1
Δrv2402

Supplementary Figure 5. Contribution of trehalose metabolism remodeling to Mtb PLB and persister formation.

a. Time-course of mRNA transcript levels of genes involved in trehalose metabolism (black), TMM biosynthesis (green), and TDM biosynthesis (red) while forming PLB within mycobacterial biofilm culture.

b. The essentiality of TreS activity for PLB formation was monitored by CV staining using wildtype Mtb (WT), *treS* deficient Mtb (ΔtreS), and ΔtreS complemented with *treS* (ΔtreS/com). The specificity of TreS activity for PLB formation was tested by including CV staining experiments of the PLB fraction using *otsB1* deficient Mtb (ΔotsB1) and *rv2402* deficient Mtb (Δrv2402).

c. Planktonic growth of WT, ΔtreS, and ΔtreS/com in Sauton nutrient-minimal media that contained either 0.2% dextrose (DEX), glycerol, or acetate (ACE) as a single carbon source was monitored by OD_{595}.

d. Killing curves of WT, ΔtreS, and ΔtreS/com following treatment with 100 µg/mL d-cycloserine. Viability was monitored by CFU/mL at 1, 7, and 14 days post-treatment. Viable colonies after a 14-day incubation are considered Mtb persisters. *, P<0.01 by ANOVA between WT and ΔtreS with Bonferroni post-test correction.

e. Metabolomics profiles of WT, ΔtreS, ΔotsB1, and Δrv2402 harvested at day 28 PLB. Analyses were focused on trehalose, maltose, and glucose 6-phosphates (G6P). Source data are provided as a Source Data file.

All values are the average of biological triplicates ± s.e.m.

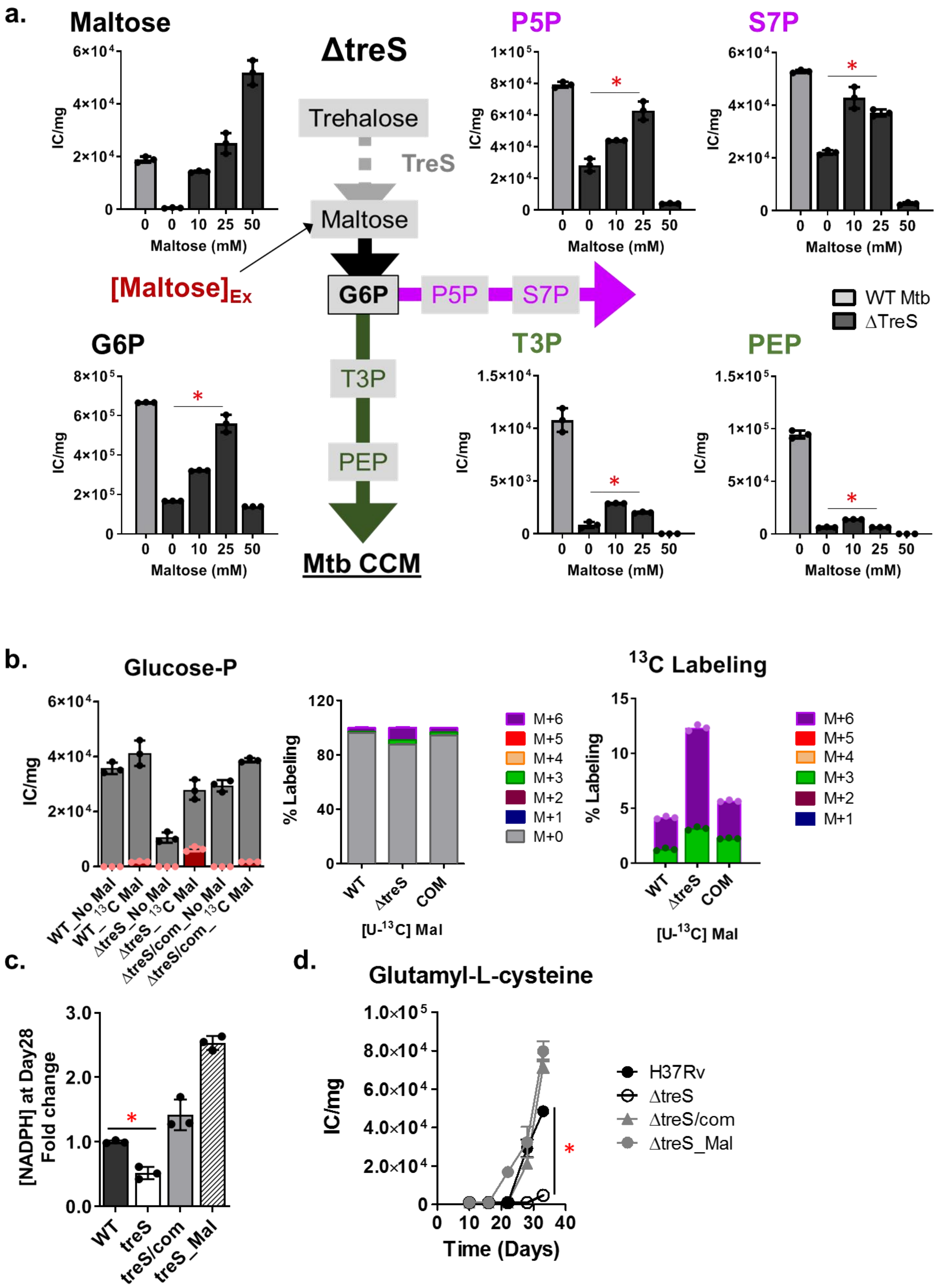
a.
Maltose
ΔtreS
P5P
S7P
Trehalose
TreS
Maltose
[Maltose]Ex
G6P
T3P
PEP
Mtb CCM
WT Mtb
ΔTreS
IC/mg
Maltose (mM)
b.
Glucose-P
13C Labeling
% Labeling
[U-13C] Mal
M+6
M+5
M+4
M+3
M+2
M+1
M+0
WT_No Mal
WT_13C Mal
ΔtreS_No Mal
ΔtreS_13C Mal
ΔtreS/com_No Mal
ΔtreS/com_13C Mal
WT
ΔtreS
COM
c.
[NADPH] at Day28 Fold change
WT
treS
treS/com
treS_Mal
d.
Glutamyl-L-cysteine
Time (Days)
H37Rv
ΔtreS
ΔtreS/com
ΔtreS_Mal

e.

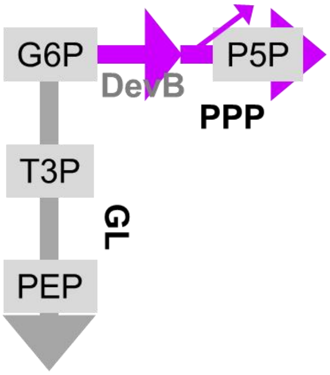
NADPH
G6P
DevB
P5P
PPP
T3P
GL
PEP

f.

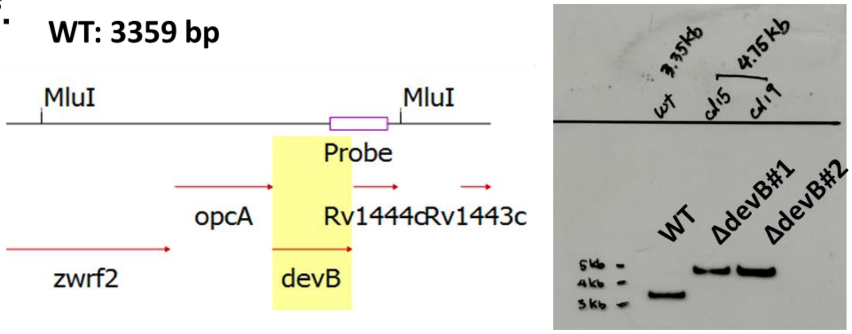
WT: 3359 bp
MluI
MluI
Probe
opcA
Rv1444c
Rv1443c
zwrf2
devB
WT
ΔdevB#1
ΔdevB#2

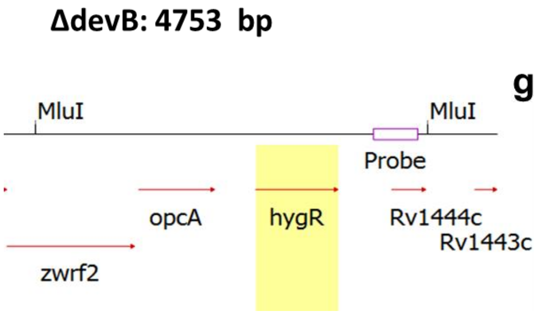
ΔdevB: 4753 bp
MluI
MluI
Probe
opcA
hygR
Rv1444c
Rv1443c
zwrf2

g.

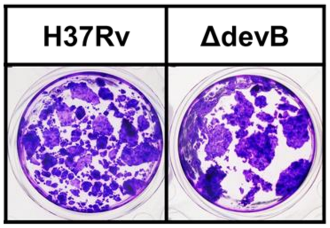
H37Rv
ΔdevB

Supplementary Figure 6. Maltose-metabolic rescue of the ΔtreS PLB fraction.

a. The effect of supplementing with 20 mM maltose on the ΔtreS PLB metabolome. The analyses were focused on intermediates in trehalose metabolism (black font/line), glycolysis (green font/line) and the pentose phosphate pathway (red font/line). Total bar heights indicate intrabacterial pool sizes of ΔtreS metabolites with or without maltose supplementation or WT Mtb metabolites. The metabolome was sampled at day 28 within mycobacterial biofilm culture. *, $P<0.001$ by Student's unpaired t-test. P5P, pentose 5-phosphates; S7P, sedoheptulose 7-phosphates; G6P, glucose 6-phosphates; T3P, triose 3-phosphates (dihydroxyacetone phosphate and glyceraldehyde 3-phosphate); PEP, phosphoenolpyruvate.

b. Intrabacterial pool size and % isotopic labeling of glucose-P extracted from WT Mtb, ΔtreS, and ΔtreS/com at day 28 of mycobacterial biofilm culture following treatment with 20 mM of a mixture of 90% ^{12}C maltose and 10% universally [U-^{13}C] maltose. Total bar heights indicate relative intrabacterial glucose-P concentration. The red area of each bar denotes the enrichment of ^{13}C labeling achieved from $^{13}C+^{12}C$ maltose mixture (left panel). Isotopologue distribution of glucose-P from the ΔtreS PLB was depicted with inclusion of M+0 isotopologue (middle panel) and exclusion of M+0 isotopologue (right panel). Source data are provided as a Source Data file.

c. The effects of TreS deficiency or exogenous supplementation with 20 mM maltose on NADPH levels of ΔtreS PLB fraction. *, $P<0.001$ by Student's unpaired t-test.

d. Time-course of glutamyl-L-cysteine levels within the PLB of WT Mtb, ΔtreS and ΔtreS/com and the effect of supplementing ΔtreS with 20mM maltose. *, $P<0.001$ by ANOVA between WT and ΔtreS with Bonferroni post-test correction.

All values are the average of biological triplicates ± s.e.m.

e. DevB is involved in second step of the pentose phosphate pathway.

f. Generation of *devB* deficient Mtb (ΔdevB). Genomic organization and Southern blot analysis design with native *devB* locus before and after replacement with a hygromycin resistance cassette. A 1 kb probe spanning Rv1444 and *devB* detects *Mlu*I-digested DNA fragments either containing the *devB* locus or *devB* elsewhere in the genome. Southern blot showing the expected band patterns for WT and two ΔdevB candidates (cd15 and cd19). WT showed a 3359 bp band indicative of an intact native *devB* locus and ΔdevB mutant strains harbor a new expected band pattern of 4753 bp.

g. CV staining pattern of PLB using WT and *devB* deficient Mtb (ΔdevB) at day 22.

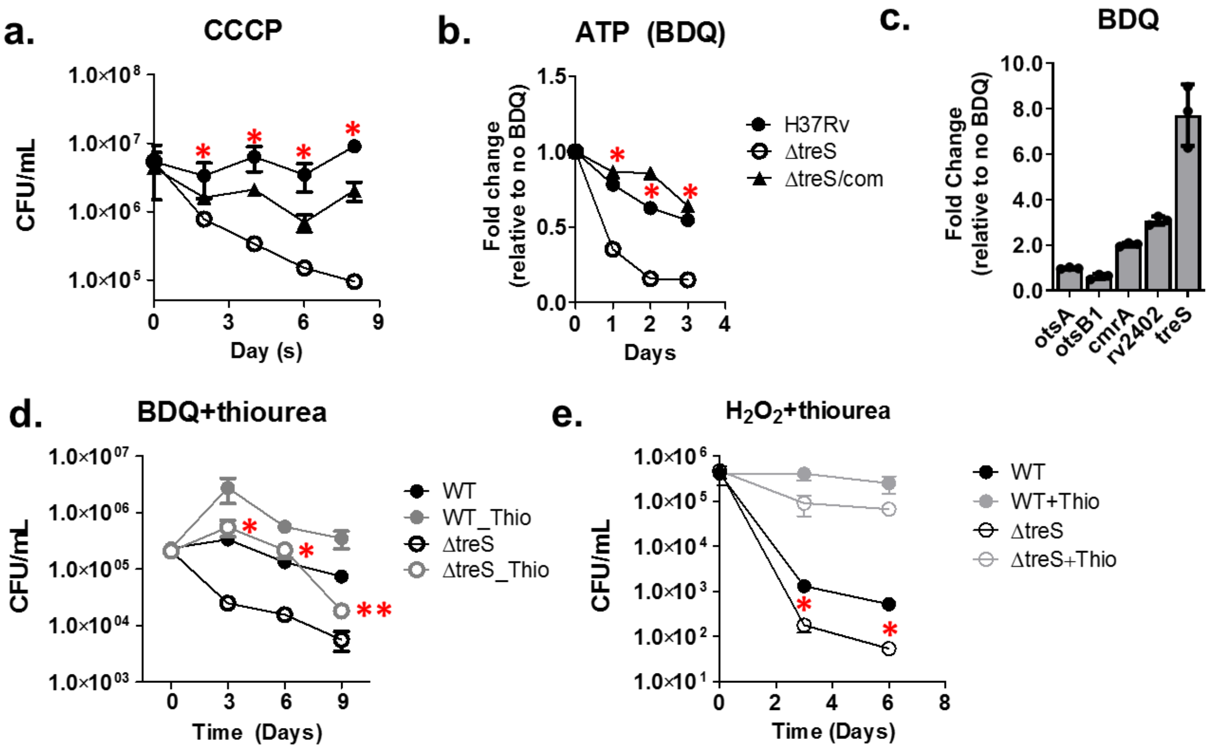

Supplementary Figure 7. Trehalse-catalytic shift serves intrinsic drug-tolerance of Mtb.

a. WT Mtb, ΔtreS, and ΔtreS/com CFU viability following treatment with 10X MIC (20 µg/mL) equivalent CCCP (ΔΨm destabilizer) for 8 days.

b. Time-course of intrabacterial ATP levels of WT Mtb ($H_{37}Rv$), ΔtreS, and ΔtreS/com following treatment with 10X MIC equivalent (0.3 µg/mL) of BDQ. *, P<0.005 by Student's unpaired t-test (between ΔtreS and WT).

c. mRNA expression level of genes involved in Mtb trehalose metabolism including *otsA*, *otsB1*, *cmrA*, *rv2402*, and *treS* following treatment with 10X MIC equivalent of BDQ. Signals were normalized to *sigA* transcript and quantified by the ΔCt method. Source data are provided as a Source Data file.

d. The effect of 25 mM thiourea on CFU-based viability of WT Mtb and ΔtreS following co-treatment with 10X MIC (0.3 µg/mL) equivalent of BDQ was monitored for 9 days.

e. The effect of 25 mM thiourea on CFU-based viability of WT Mtb and ΔtreS following 5 mM H_2O_2 as a ROS inducer. CFU was monitored for 6 days. Thio, thiourea. *, P<0.001; **, P<0.01 by Student's unpaired t-test (between ΔtreS and ΔtreS_Thio).

All values are the average of experimental triplicates ± s.e.m.

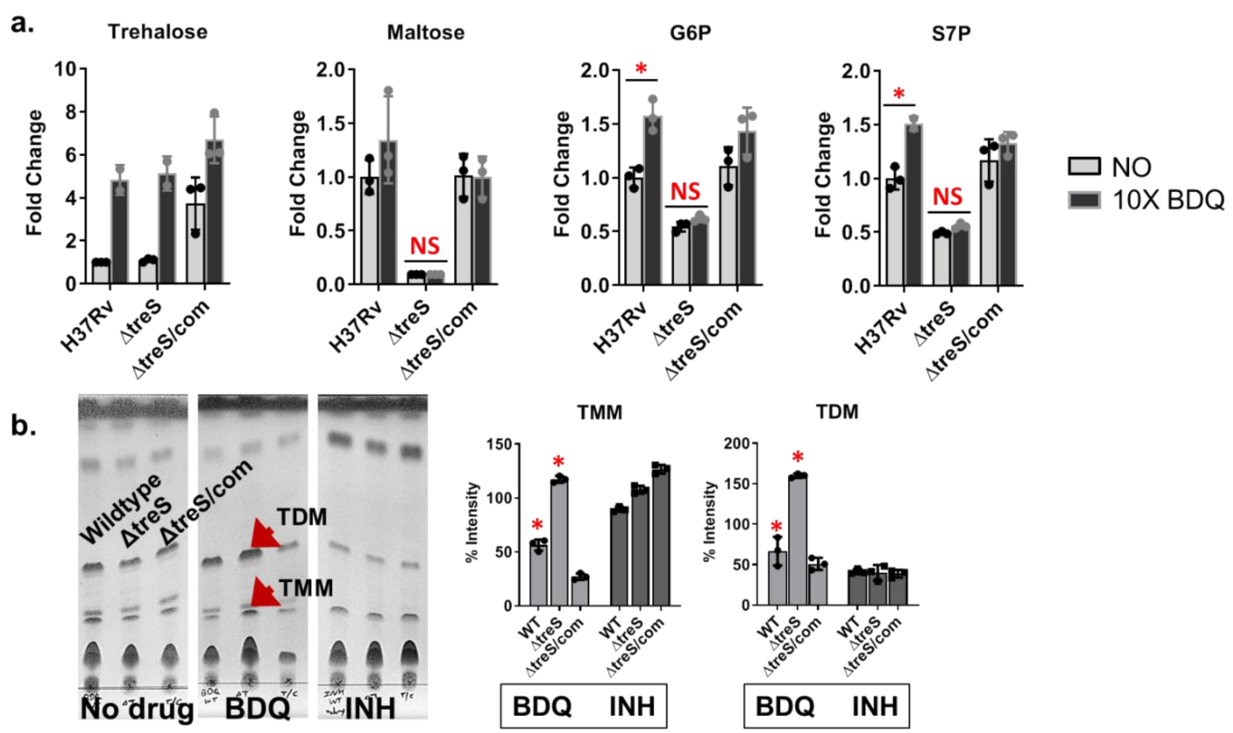

Supplementary Figure 8. The effect of TreS deficiency on the Mtb metabolome following treatment with BDQ.

a. Intrabacterial pool sizes of WT Mtb ($H_{37}Rv$), ΔtreS, and ΔtreS/com intermediates in trehalose metabolism (trehalose and maltose), glycolysis (G6P) and PPP (S7P) following treatment with 10X MIC equivalent of BDQ. *, P<0.01; NS, not significant by Student's unpaired t-test. Glucose phosphates, G6P; Sedoheptulose phosphates, S7P. Source data are provided as a Source Data file.

b. TLC-based lipid profiles of WT Mtb, ΔtreS, and ΔtreS/com TMM and TDM developed using the chloroform: methanol: H_2O (90:10:1, v:v:v) solvent system and visualized by charring with 1% molybdophosphoric acid. Red arrow heads indicate accumulated TMM and TDM in ΔtreS following treatment with 10X MIC equivalent of BDQ as compared to those of WT Mtb or ΔtreS/com (middle panel). No drug-treatment (left panel) and 10X MIC equivalent of INH (right panel) were included as controls. TMM/TDM intensity (density) of Mtb following drug treatment was quantified by ImageJ software. *, P<0.05 by Student's unpaired t-test (changes relative to counterparts under no drug treatment). TMM, trehalose monomycolate; TDM, trehalose dimycolate.

All values are the average of biological triplicates ± s.e.m.

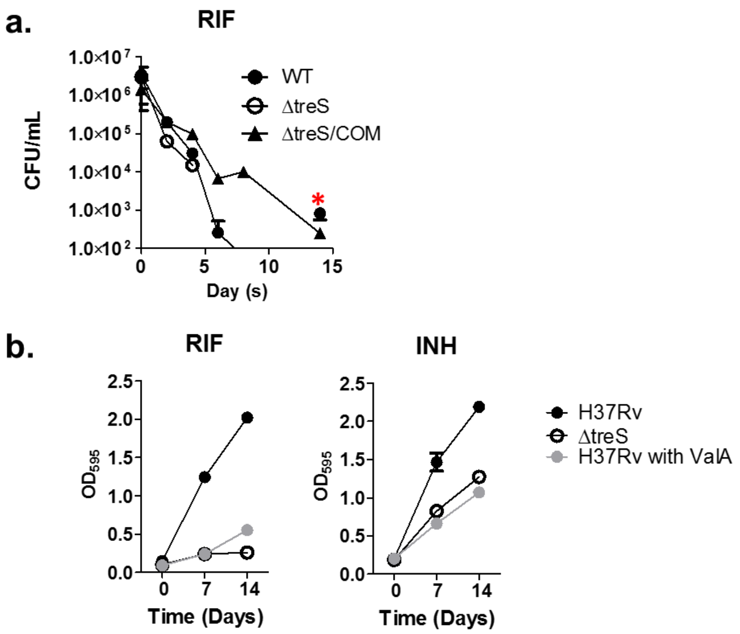

Supplementary Figure 9. Role of trehalose-catalytic shift in VBNC formation following first-line TB drugs.

a. CFU-based viability of WT Mtb, ΔtreS, and ΔtreS/com following treatment with 10X MIC equivalent amount of rifampicin (RIF). *, P<0.01 by Student's unpaired t-test between $H_{37}Rv$ and ΔtreS.

b. The effect of TreS deficiency on Mtb outgrowth following treatment with 30X MIC equivalent amount of rifampicin (left panel) or isoniazid (right panel) for 10 days. TreS deficiency was obtained using either ΔtreS (genetic inactivation) or WT Mtb with validamycin A (Val A) (chemical inactivation). Standard outgrowth assays were conducted to estimate the number of viable bacteria that lacked the ability to form colonies (VBNC, viable but non-culturable).

All values are the average of biological triplicates ± s.e.m.

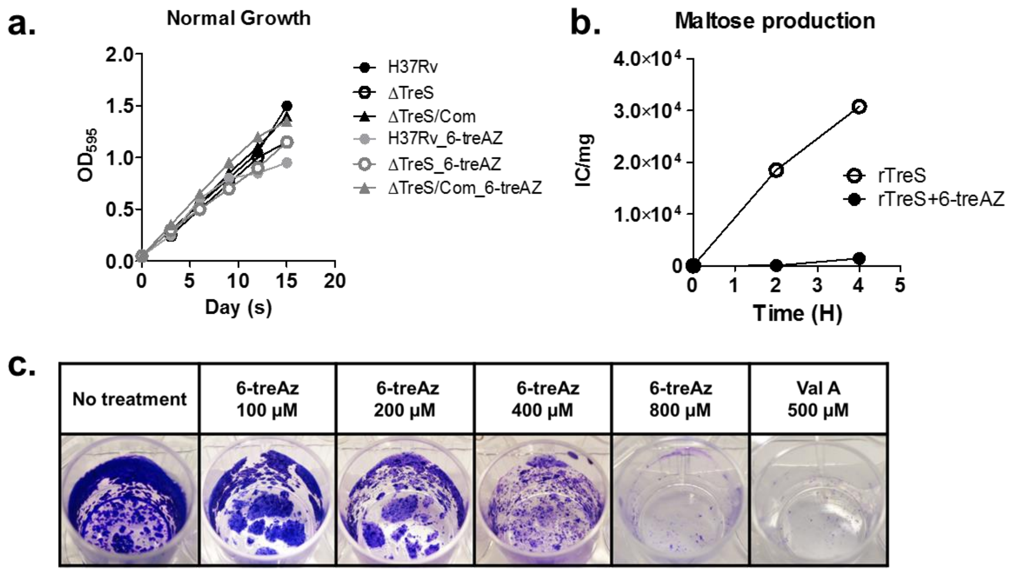

Supplementary Figure 10. Characterization of 6-Azido-6-α,α'-trehalose (6-treAz) roles.

a. The effect of 400 µM 6-treAz treatment on Mtb planktonic growth using Sauton nutrient-minimal media. At or above 800 µM 6-treAz, Mtb planktonic growth was interfered.

b. The effect of 6-treAz on TreS-mediated trehalose to maltose conversion was monitored using an *in vitro* enzyme reaction. *In vitro* TreS reactions contained 0.1 µg of recombinant TreS, 10 mM $MgCl_2$, 0.1 mM trehalose in the presence or absence of 100 µM 6-treAz, incubated at 37 °C for up to 4 hrs. Maltose production was monitored by LCMS.

c. CV staining pattern of WT Mtb PLB following treatment with various concentrations (0 – 800 µM) of 6-treAz or 500 µM validamycin A (Val A). The PLB was harvested at day 22 of mycobacterial biofilm culture.

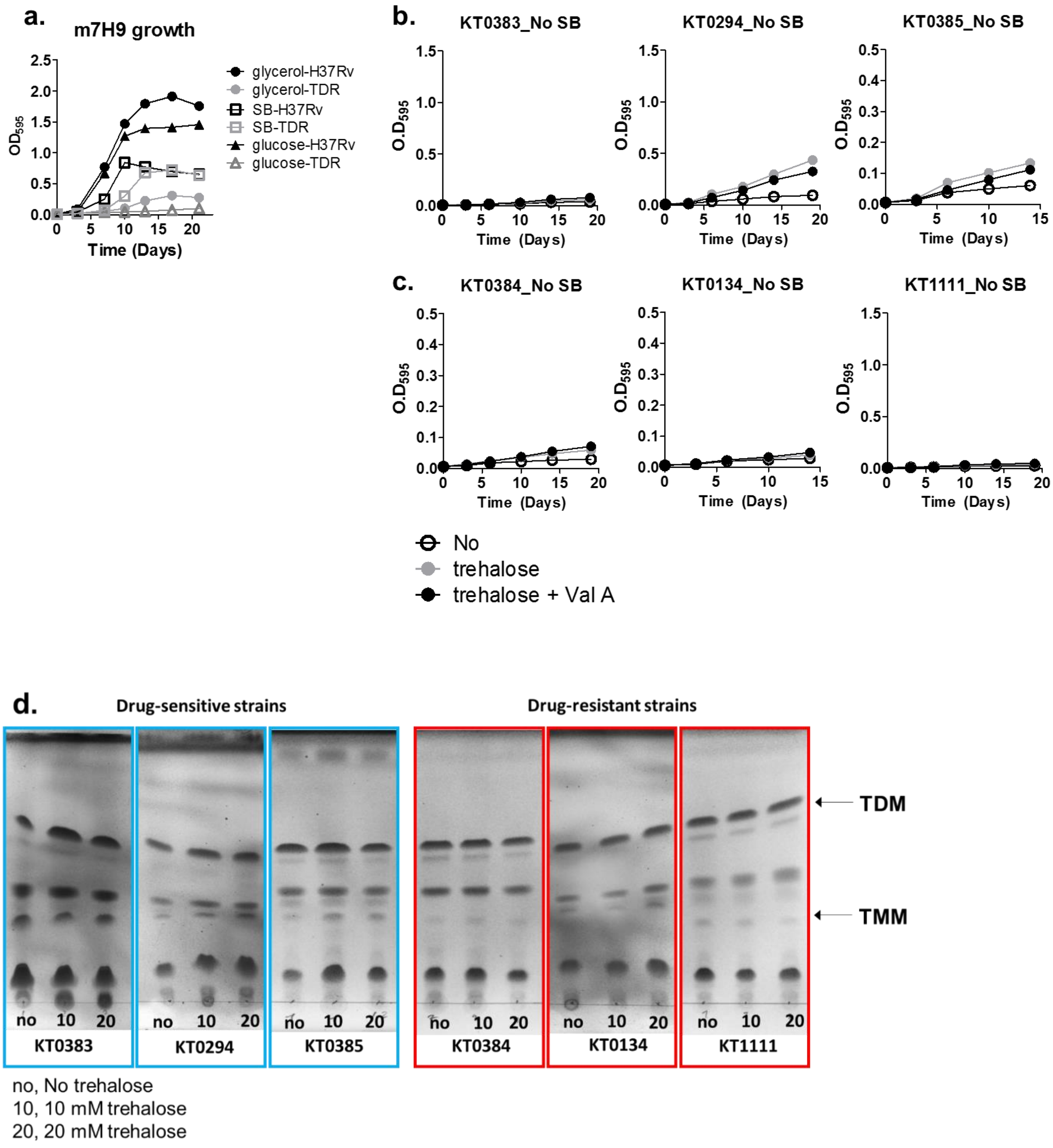
a.
m7H9 growth
glycerol-H37Rv
glycerol-TDR
SB-H37Rv
SB-TDR
glucose-H37Rv
glucose-TDR
Time (Days)
b.
KT0383_No SB
KT0294_No SB
KT0385_No SB
c.
KT0384_No SB
KT0134_No SB
KT1111_No SB
Time (Days)
No
trehalose
trehalose + Val A
d.
Drug-sensitive strains
Drug-resistant strains
TDM
TMM
KT0383
KT0294
KT0385
KT0384
KT0134
KT1111
no, No trehalose
10, 10 mM trehalose
20, 20 mM trehalose

Supplementary Figure 11. The characterization of XDR- and TDR- TB clinical isolates.

a. Growth of the TDR (KT1111)-TB isolate in m7H9 media containing either 0.2% sodium butyrate, 0.2% glycerol, or 0.2% glucose was compared to that of the lab strain ($H_{37}Rv$). Only 0.2% sodium butyrate (SB) carbon supported TDR-TB isolate growth at levels comparable to that of $H_{37}Rv$.

b. The growth of drug-sensitive isolates in m7H9 media containing 20 mM trehalose in the presence or absence of 200 µM validamycin A (Val A) was monitored by OD_{595}.

c. The growth of drug-resistant isolates (two XDR-TB isolates and one TDR-TB isolate) in m7H9 media containing 20 mM trehalose in the presence or absence of 200 µM Val A was monitored by OD_{595}.

All values are the average of biological triplicates ± s.e.m.

d. The effect of exogenous supplementation with 10 and 20 mM trehalose on TMM/TDM abundance of drug-sensitive (KT0294, KT0383, and KT0385) and drug-resistant (KT0134, KT0384, and KT1111) TB clinical isolates was resolved by TLC-based lipid profile.

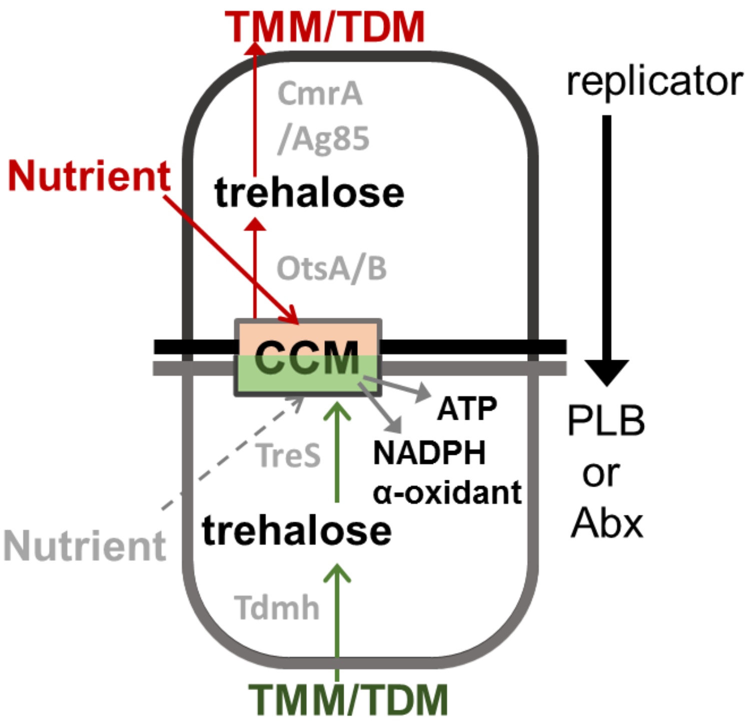

Supplementary Figure 12. Schematic diagram of PLB specific trehalose-catalytic shift

Mtb in a replicating state (top half, black) actively consumes external nutrient and thereby biosynthesize CCM intermediates, with which trehalose and cell surface TMM/TDM are biosynthesized via OtsA/B, CmrA, and Ag85 activity. In response to environmental stresses including antibiotic (Abx) treatment or PLB formation (down half, gray), Mtb ceases uptake/consumption of external nutrient. Instead, Mtb uses free trehalose or trehalose released from dissociated TMM/TDM to maintain CCM activities, as alternate ATP, NADPH and antioxidant sources. Tdmh was shown to be a putative activity required for TDM hydrolysis [35]. CCM, central carbon metabolism.

Supplementary Table 1. Primers used for qRT-PCR

Primer	Name	Sequence (5' - 3')
1	SigA-F<br>SigA-R	ACGAAGACCACGAAGACCTCGAA<br>GTAGGCGCGAACCGAGTCGGCGG
2	TreS-F<br>TreS-R	CGGTGACTATTACGTGTGGAG<br>GAAGAATCGGTGCCAGTAGAA
3	OtsA-F<br>OtsA-R	GTTGGCGCCTTTCCTATCT<br>AGGATCTTGCGCGGATTT
4	OtsB1-F<br>OtsB1-R	AATTGGTCGGGCACAGTT<br>GGTAAGGTGTTGGCTGGATAG
5	OtsB2-F<br>OtsB2-R	GCAGCCATGGTTTCGAATTG<br>TGTTTCAGCACCGGTATGG
6	CmrA-F<br>CmrA-R	ACGCAGGTGCAGTTGAAT<br>CGTTGTAGGGAATCGGTGAAT
7	LAT-F<br>LAT-R	CTGGACATAGTGCTCGATCTG<br>AGGAGGCAACGAATGTGAA
8	Rv2402-F<br>Rv2402-R	CAACAACAACGAACGCCATC<br>CATGATCGTAGCCGGACAAA
9	Pks13-F<br>Pks13-R	CCTACTTCAAGGACCACCTCTA<br>GAGATCCTGTGGGTAGTGATTG
10	FbpA (Ag85a)-F<br>FbpA (Ag85a)-R	TACAAGTGGGAGACCTTCCT<br>AAGAAGCAGCCATCGAAAGA
11	FbpB (Ag85b)-F<br>FbpB (Ag85b)-R	CACCCGGCTATGGGTTTATT<br>GCTGCTACGAACGAAGTTCT
12	FbpC (Ag85c)-F<br>FbpC (Ag85c)-R	CAGTTTCTACACCGACTGGTATC<br>TCTCTCTGGTAAGGAAGGTCTC
13	Rv2667-F<br>Rv2667-R	GCTCATCAACGCCATCAAAC<br>GACGAAGTGGCCGATTAAGT
14	Rv3287c-F<br>Rv3287c-R	AAGACGAAGTTGTGGTGGAG<br>TCAGGACATGCCAGCTAAAG
15	Rv3286c-F<br>Rv3286c-R	GGTCTTGACCAGATCGAGAATC<br>GAAGAACCTGAGCACCAAGA
16	Rv2651c-F<br>Rv2651c-R	CTGTTGGAGGTCTCCTTGAC<br>ACCAATCCATCAGGGCTAAC
17	Rv0840c-F<br>Rv0840c-R	CGTAGACCTTTGGACGATGAA<br>CGAATGGCTGAAGATGTGAAAG
18	Rv2662-F<br>Rv2662-R	CTCCGACTCGTGCGATTAAC<br>TTCTCACCAGCGCAAACA
19	Rv2666-F<br>Rv2666-R	TGCTACCTGCTGGGAGTAT<br>TGATCGACACTTGCGACTTG
20	Rv0792c-F<br>Rv0792c-R	CCCTTGAAGTGGTCGTGTT<br>CGATAATTCCGGCACCTGAT
21	Rv0142-F<br>Rv0142-R	TTGGTGTCCAAGTACGGAAC<br>GACATTGGCGCGATGAAAC
22	Rv0837c-F	GGTGGCCTATCAGCATTGAA

	Rv0837c-R	CGACAGGTGAGGAAGGTAGA
23	Rv2661c-F<br>Rv2661c-R	CATCAGTGCCCTCGTTGATAAT<br>CTCGGCCTGTCCACAAAC
24	Rv3370c-F<br>Rv3370c-R	ATGCTATGCGCTGTCGTT<br>TCGCCGCAATCAAGAGATATT
25	Rv0849-F<br>Rv0849-R	GTTGTTCGAGCGCTTCTTATC<br>GTAGACGTACTGGATGACCTTT
26	Rv1707-F<br>Rv1707-R	CCACGGTCCACAGTGAAAT<br>GACAACCCACGACCATCAG
27	Rv2623-F<br>Rv2623-R	GGAATCGCGATCCTCATCAT<br>CGGTGGAATCGCTTTGATAAAC
28	Ndh-F<br>Ndh-R	GTTGAGTTCAGCGGCTTTATC<br>GACAGCAGAGTGGTGATCTT
29	NouG-F<br>NouG-R	CGGTCAGCTATGCCGATTT<br>TGCGTAACCGCAGAAACA
30	FdxA-F<br>FdxA-R	GCCCGAATGCTCTACATCAA<br>AGATCGCCTTCCCAGTAGAT
31	FdxD-F<br>FdxD-R	GGAATCGCACCGGATATCTT<br>GCCAGATCCTCCTGATCAAC
32	PfkB-F<br>PfkB-R	TGGCTGCCGACTACTATCA<br>GCTCGCCTTGAGAAGAAACA
33	Pgi-F<br>Pgi-R	GGACTGTGGTACTCCAATTTCT<br>ATGGTCAACTGCTGAAGGTAG
34	Pgk-F<br>Pgk-R	CTGCTGGAAGACGACATGAT<br>ACTTCTCCGTGACCACTAGAT
35	PykA-F<br>PykA-R	CGGAAGCCATCGACAATCT<br>TACCAGCGGGACCTCTT
36	Gap-F<br>Gap-R	GGAGTTAACGACGACAAGTATGA<br>TCATCGTCGAGCACTTTGG
37	AtpB-F<br>AtpB-R	GGTGCAGTACACCGATAAACA<br>GGTAGCAGACGAACACGAAA
38	AtpF-F<br>AtpF-R	TCGCTGTCATTGGCACTT<br>CTCGTCCGACTTCTTGTTGT
39	Ace-F<br>Ace-R	ATCGCCTACATCGTGGAAAG<br>CTCGTTGTAGACGGTGATGTAG
40	BkdA-F<br>BkdA-R	GAATTGGGCGTCTACCTAGTG<br>ACAGCACTTCGTGGTGAAT
41	TPI-F<br>TPI-R	GGAAGATGAACCTCAACCACTA<br>GTCAACCCGGTCGTAATACTT
42	RpmB1-F<br>RpmB1-R	CACTGTGGGTTTCGGTAAGG<br>GTAGGCCTTGAGCTGGATATTG
43	DosR-F<br>DosR-R	CCGAATGTTCCTAGCCGAAA<br>TTCAACTCCGTCGCGAATAC
44	Rv2402-F<br>Rv2402-R	CAACAACAACGAACGCCATC<br>CATGATCGTAGCCGGACAAA
45	DnaE1-F<br>DnaE1-R	GATCTCTTCGGCAGCAATGA<br>CTCTAGGGCGAGTTTGTGTTT

46	GlnA1-F<br>GlnA1-R	CGGCCAAGACGCTGAATA<br>CAGTGCTGATCAGGTAGTTCTC
47	GltA2-F<br>GltA2-R	GAGGTCTGCTACCTGTTGATTTA<br>GTCGAAGAACCGCTTGAGAT
48	GlcB-F<br>GlcB-R	CGACAAGGTGCTGGGTTAT<br>GTGTAGTTCCGGTCCCTATTG
49	Icd1-F<br>Icd1-R	CGCGAACCTATCGTGATTTCTA<br>GAAGTTCGTCGCCCGATATT
50	Icd2-F<br>Icd2-R	ACCCAAGCACGTCAAACA<br>TGATGCCGATATCCTCGAAAC
51	Icl1-F<br>Icl1-R	ATCGCCAAGTTCCAGAAGG<br>GCCAGATCGAACATCGAGTAG
52	Icl2-F<br>Icl2-R	CGACTTCCGCCCGTTTAT<br>GTTGGTCCTCGATGTGGTAG
53	PrpC-F<br>PrpC-R	GCGTACTACCTGATGGGATTC<br>CGTGGCCTGTTCCATGAT
54	PrpD-F<br>PrpD-R	GCACTGTGGCACAAGATTTC<br>TTCGTCCACGATCACTTCAC
55	PrpR-F<br>PrpR-R	AACTAGAGGGCTACCGATCTC<br>GTAGTTGTTGCGGTCGTAGAA
56	MmpL3-F<br>MmpL3-R	CGACCTGGTGATGATGAAGTATC<br>CTTCATCACCGATGGCACTAA

Supplementary Table 2. Information of clinical TB isolates used in this study

Drug-susceptibility test (Löwenstein–Jensen medium)																
	INH	RIF	SM	EMB	KM	CPM	PTH	DCS	PAS	OFX	MFX	AMK	LEV	RBU	PZA	PNB
KT 0383	S	S	S	S	S	S	S	S	S	S	S	S	S	S	S	S
KT 0294	S	S	S	S	S	S	S	S	S	S	S	S	S	S	S	S
KT 0385	S	S	S	S	S	S	S	S	S	S	S	S	S	S	S	S
KT 0384	R	R	S	R	S	S	R	R	S	R	R	S	R	S	R	S
KT 0134	R	R	S	R	R	S	R	R	R	R	S	S	R	S	S	S
KT 1111	R	R	R	R	R	R	R	R	R	R	R	R	R	R	R	R

MGIT culture										
	SM	INH	RFP	EMB	KM	CPM	KM	OFX	MFX	PZA
KT 0383	S	S	S	S	S	S	S	S	S	S
KT 0294	S	S	S	S	S	S	S	S	S	S
KT 0385	S	S	S	S	S	S	S	S	S	S
KT 0384	S	R	R	S	S	S	R	R	R	S
KT 0134	S	R	R	S	S	R	R	R	S	S
KT 1111	R	R	R	R	R	R	R	R	R	R

Abbreviation: INH, isoniazid; RIF, rifampicin; SM, streptomycin; EMB, ethambutol; KM, kanamycin; CPM, capreomycin; PTH, prothionamide; DCS, d-cycloserine; PAS, p-aminosalicylic acid; OFX, ofloxacin; MFX, moxifloxacin; AMK, amikacin; LEV, levofloxacin; RBU, rifabutin; PZA, pyrazinamide; PNB, para-nitrobenzoic acid; TH, ethionamide. S, sensitive; R, resistant.
